# Mediating role of television time, diet patterns, physical activity and sleep duration in the association between television in the bedroom and adiposity in 10 year-old children

**DOI:** 10.1186/s12966-015-0221-5

**Published:** 2015-05-13

**Authors:** Michael M Borghese, Mark S Tremblay, Peter T Katzmarzyk, Catrine Tudor-Locke, John M Schuna, Geneviève Leduc, Charles Boyer, Allana G LeBlanc, Jean-Philippe Chaput

**Affiliations:** Healthy Active Living and Obesity Research Group, Children’s Hospital of Eastern Ontario Research Institute, 401 Smyth Road, Ottawa, K1H 8L1 ON Canada; School of Human Kinetics, Faculty of Health Sciences, University of Ottawa, Ottawa, ON Canada; Department of Pediatrics, Faculty of Medicine, University of Ottawa, Ottawa, ON Canada; Population Health, Faculty of Graduate and Postdoctoral Studies, University of Ottawa, Ottawa, ON Canada; Pennington Biomedical Research Center, Baton Rouge, LA USA

**Keywords:** Diet, Lifestyle habits, Mediation, Obesity, Screen-based media

## Abstract

**Background:**

Having a TV in the bedroom is associated with adiposity in children. It is not known how lifestyle behaviours (television viewing time, diet patterns, physical activity, and sleep duration) mediate this association. The objective of this study was to examine the mediating role of these lifestyle behaviours in the association between TV in the bedroom and percent body fat (% BF).

**Methods:**

Cross-sectional data from 1 201 children (57.3 % female; mean age = 9.8 years) from Ottawa, Canada and Baton Rouge, USA were examined. % BF was directly measured. Accelerometers were used to determine physical activity and sleep duration (24-h, 7-day protocol). Questionnaires were used to assess TV viewing time and healthy/unhealthy diet patterns (derived using factor analysis from food frequency questionnaire data).

**Results:**

Canadian boys and girls with a TV in their bedroom had a higher % BF, watched more TV and had unhealthier diets. American boys and girls with a TV in their bedroom watched more TV, while boys had a higher % BF and a more unhealthy diet, and girls had less MVPA. In Canadian girls, TV viewing time mediated the association between having a TV in the bedroom and adiposity, independent of diet patterns, MVPA, and sleep duration. Other lifestyle mediators were not significant in Canadian boys or in US children.

**Conclusion:**

TV viewing is a mediating lifestyle behaviour in the association between TV in the bedroom and adiposity in Canadian girls. Future research is needed to identify lifestyle behaviours as intermediate mediators.

## Introduction

Young people now spend more time with media than they do in school and, other than sleeping, TV viewing is the leading activity for children and adolescents [1]. Previous work has shown a clear link between increased TV viewing time and poor health indicators in children and youth, including decreased fitness, lowered scores for self-esteem and pro-social behaviour as well as unfavorable body composition [2]. The omnipresence of screens (especially TV) in children’s lives poses a potential health risk, and society as a whole has a role to play to mitigate these risks. Interventions that may be capable of addressing this risk by reducing children’s screen time are being tested [3, 4].

Recent estimates suggest that 71 % of American children and adolescents have a TV in their bedroom [5]. The American Academy of Pediatrics suggested in 2013 that parents should remove TVs from children’s bedrooms (along with internet connected electronic devices), thereby reducing their access [6]. This is largely because evidence suggests that the presence of a TV in children’s bedrooms increases TV viewing time [7], risk of substance use, and adiposity [6, 8], as well as other health risks. While it may seem intuitive that bedroom TVs exert their effect on children’s adiposity through increased TV viewing time, there is evidence to suggest that this effect exists beyond that which can be explained by TV viewing time alone [9, 10]. Having a TV in the bedroom is also associated with unhealthy food choices [11], lower levels of moderate-to-vigorous physical activity (MVPA) [12], and poor sleep habits [13, 14] in children. However, this evidence is mixed [10], and the mechanisms behind the association between TV in the bedroom and adiposity in children are unclear.

Previous studies have focused primarily on the association of bedroom TVs and a specified outcome measure without considering potential mediating or moderating effects of other factors, despite the fact that these variables are generally interrelated. We are aware of two other studies that have examined the potential mediating effects of children’s lifestyle behaviours on the association between having a TV in the bedroom and adiposity. However, neither of these studies used objective measures of physical activity and sleep duration, and both used BMI as a measure of obesity instead of adiposity [7, 15]. While these analyses provide many insights, the literature is limited in examining the influence of TV in the bedroom on diet patterns in children; studies have examined the link with sugar-sweetened beverage consumption [7, 11], but the association with children’s habitual diet patterns has not been addressed. Thus, a comprehensive evaluation of the potential associations between having a TV in the bedroom and lifestyle behaviours using objective measures is warranted.

Accordingly, the purpose of this study was to examine the association between having a TV in the bedroom and percent body fat (% BF) in both Canadian and American children, considering the following lifestyle behaviours as mediating factors: TV viewing time, diet patterns, MVPA, and sleep duration. It was hypothesized that children with a TV in their bedroom would have higher % BF and total TV viewing time, poorer diet patterns, lower MVPA, and shorter sleep duration than those with no TV in their bedroom. It was also hypothesized that the aforementioned lifestyle behaviours would independently mediate the association between TV in the bedroom and % BF in children. It is crucial that mediation analyses using cross-sectional data have a strong theoretical/conceptual foundation. The conceptual model for the mediating role of lifestyle behaviours in the association between having a TV in the bedroom and adiposity in children is based on previous evidence from longitudinal [9 ,10, 14, 16, 17] and intervention studies [18, 19]. Furthermore, this mediation approach has previously been used with cross-sectional data to answer similar research questions [15, 20]. While cross-sectional mediation analysis can be informative in the context of existing conceptual models, potential mediating factors should not be interpreted as being causal, but rather informative for further hypothesis generation.

## Methods

### Participants

The International Study of Childhood Obesity, Lifestyle and the Environment (ISCOLE) is a multi-national, cross-sectional study conducted in 12 countries. The primary purpose of ISCOLE is to construct a statistical model which can predict adiposity in children based on dietary habits and physical activity, as well as other environmental variables. Data from the NHANES 2005/2006 informed an a priori power calculation which indicated that a sample size of 500 participants from each of the 12 international sites would allow for statistical power of 97 %, when alpha = 0.05 and variance in adiposity (R^2^) explained by either dietary habits or physical activity = 3 %. The targeted overall sample included 6000 10-year-old children from 12 countries in five major geographic regions of the world (Europe, Africa, the Americas, South-East Asia, and the Western Pacific). Further details pertaining to the study design and methods can be found elsewhere [21]. Analyses herein include data from the Canadian and American ISCOLE sites.

In Canada, data were collected in 26 schools on 567 children (mean age = 10.0, 57.8 % female) from Ottawa, Ontario between September 2012 and May 2013. Schools were stratified into four groups: 1) English Public (n = 393; 69.3 %), 2) French Public (n = 60; 10.6 %), 3) English Catholic (n = 75; 13.2 %), and 4) French Catholic (n = 39; 6.8 %). In the United States, data were collected in 21 schools on 651 children (mean age = 9.5, 56.8 % female) from Baton Rouge, Louisiana between August 2012 and May 2013. Schools were stratified into 5 groups: 1) Public – 95.0-100 % on free and reduced price meals (n = 142; 21.8 %), 2) Public – 85.5-95.0 % on free and reduced price meals (n = 44; 6.8 %), 3) Public – 73.4-85.5 % on free and reduced price meals (n = 115; 17.7 %), 4) Public – 1.4-73.4 % on free and reduced price meals (n = 181; 27.8 %), and 5) Private (n = 169; 26.0 %). The cities from each country were selected based on their proximity to the country-specific study site, and the data collected are not intended to form a representative sample of the country or region specific to the study site. At both the Canadian and US sites, schools within each stratum were invited to participate and the first to respond were enrolled in the study, in agreement with the local research ethics or institutional review boards. In all schools children (and their parents/guardians) were invited to participate on a volunteer basis. They were recruited through letters sent home to the parents. This project was approved by the research ethics board at the Children’s Hospital of Eastern Ontario (Canada) and the Pennington Biomedical Research Center (USA) as well as the participating school boards (Canadian site) or other school authority (American site). Written informed parental consent and child assent were obtained for all participants.

### Demographic information

Demographic questionnaires completed by parents were used to determine children’s age (from date of birth), sex, ethnicity (White/Caucasian, African American, Asian, First Nations, East Indian, Pacific Islander, “don’t know”, or “other”), total household annual income (4 levels based on site-specific household annual income), and the highest level of parental education (less than high school, some high school, high school diploma/GED, diploma or 1–3 years of college, bachelor’s degree, or graduate degree [master’s or PhD]/professional degree).

### Adiposity

Trained study staff collected anthropometric data in schools during school hours, following standardized procedures [21]. % BF was measured to the nearest 0.1 % using a portable Tanita SC-240 Body Composition Analyzer (Arlington Heights, IL, USA). The Tanita SC-240 showed acceptable accuracy for estimating % BF in children when compared with dual-energy X-ray absorptiometry (error = −1.0 %), supporting its use in field studies [22].

### Screen time

During the school visit, participants completed a diet and lifestyle questionnaire which included a self-reported measure of having a TV in the bedroom (“Do you have a television in your bedroom?”, with response options of “yes” or “no”). Also, children were asked how many hours/day they engaged in sedentary behaviours (TV, video games, and computers) on a typical school day and on a typical weekend day, based on a question adapted from the US Youth Risk Behaviour Surveillance System (YRBSS) [23]. The TV viewing time question derived from the YRBSS was shown to have adequate reliability with a one week test-retest interval (spearman correlation = 0.55-0.68) and validity as compared to 7-day TV time use logs (spearman correlation = 0.47) [24]. Furthermore, self-report methods of quantifying screen time have been shown to have acceptable reliability and validity in children [25]. The response options included: no use, <1 h, 1 h, 2 h, 3 h, 4 h, and ≥5 h/day. A weighted mean hours/day of TV viewing was calculated as follows: [(hours of TV on weekdays x 5) + (hours of TV on weekend days x 2)]/7. This method of determining daily amount of TV viewing has been used elsewhere [26]. This approach was also applied for determining computer and video game time (not including tablets or smartphones). Weighted means were then summed to provide an estimate of total screen time over an entire week (derived from daily screen time) for meeting or not meeting the sedentary behaviour guidelines of no more than 2 h/day [27]. All children provided data for the presence of a TV in their bedroom and their screen-based sedentary behaviours.

### Diet patterns

Children in ISCOLE were asked to complete a food frequency questionnaire (FFQ) as part of a diet and lifestyle questionnaire. The FFQ was adapted from the Health Behaviours in School-age Children study [28], and is a reliable questionnaire (test-retest reliability spearman correlation = 0.52-0.82) that can be used for ranking the frequency of consumption of most food items in children [29]. Also, the relative validity (agreement) of this FFQ was established against a food behaviour checklist and a 7-day food diary [29]. The FFQ asked the participants how often they consumed 23 food items in a usual week. There were 7 response options ranging from ‘never’ to ‘every day, more than once’. In total, 1 195 children responded to each and every one of the 23 FFQ items. This FFQ does not provide an estimate of energy intake or other indicators of the amount of food consumed. Instead, principal component analysis was used to identify diet patterns in the sample with the food items as input variables. Eigenvalues and a scree plot analysis were used to determine the appropriate number of factors. Two factors were chosen for analysis: a “healthy diet” factor and an “unhealthy diet” factor. Higher values on the healthy diet score indicate a more healthy diet, and higher values on the unhealthy diet score indicate a more unhealthy diet. These were rotated with an orthogonal varimax transformation and standardized to ensure normality.

### Physical activity and sedentary time

Time spent in MVPA was measured using the ActiGraph GT3X+ accelerometer (ActiGraph LLC, Pensacola, FL, USA) [21]. Study staff instructed children to wear the device on a belt around the waist at the right hip (mid-axillary line) 24 h/day for 7 consecutive days. Children were asked to remove the device for aquatic activities and showering/bathing. To increase wear time compliance, study staff conducted an in-school check after initialization to ensure the child was following the accelerometer wear protocol. Up to two compliance phone calls were also made to the parents/guardians (one weekday call and one weekend call) to ensure that the device was being worn properly. Data were collected at sampling rate of 80 Hz, integrated to 1-s epochs, and further aggregated to 15 s epochs for analysis [30]. MVPA was defined as all minutes showing ≥574 counts/15 s, consistent with widely used cut-points for accelerometry output [30]. Time spent in MVPA included only minutes from waking wear time (wear time minus time spent sleeping) on valid days. A valid recording required at least 4 days (including at least one weekend day) of at least 10 h of wear time per day [31, 32]. Complete and valid accelerometer data were available for 1 014 children. Children without complete accelerometry data differed in their ethnicity, total annual household income and the highest level of parental education (data not shown). These children were predominantly African American, while children with complete data were predominantly Caucasian. Further, children without complete data were more likely to live in households with lower total annual household income and where the highest level of parental education was lower than those with complete data. These variables were considered as covariates in all analyses herein.

### Sleep duration

Nocturnal sleep time was objectively assessed using the same ActiGraph GT3X+ for a 7-day period. A fully automated algorithm for 24-h waist-worn accelerometry was recently validated for ISCOLE and used for the present study [33,34]. This new algorithm captures sleep period time from sleep onset to the end of sleep, including all epochs and wakefulness after onset [33]. The weekly total sleep time averages were calculated using only days where valid sleep was accumulated (total sleep period time ≥160 min) and only for participants with at least 3 nights of valid sleep, including 1 weekend night (Friday or Saturday). Accelerometer-based sleep duration data were available from 1 008 children. Children who did not meet the criteria for valid sleep data (either missing or invalid) differed in their ethnicity, total annual household income and the highest level of parental education in the same direction as those who did not provide valid physical activity data (data not shown).

### Statistical analysis

All statistical analyses were performed using SAS 9.3 (SAS Institute, Cary NC). The school-based recruitment strategy was not accounted for in the current analysis because the majority of the variance in adiposity was explained at the individual level, rather than the school level. Furthermore, the primary variable of interest, TV in the bedroom, is a home-based characteristic which is not thought to differ by school. Independent samples t-tests for continuous variables and chi-square or Fisher’s exact tests for categorical variables (as appropriate) were used to determine significant differences in demographic characteristics and lifestyle behaviours between children who had a TV in their bedroom vs. those who did not.

**Fig. 1:**
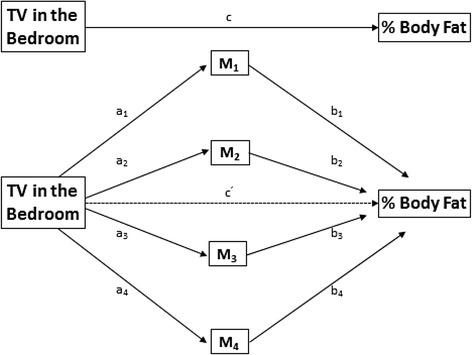
Multiple mediation on the relationship between TV in the bedroom and percent body fat through mediators. M represents the mediators, c represents the total effect of the TV in the bedroom (IV) on the % body fat (DV) and c^’^ represents the direct effect of the IV on the DV in the presence of mediators and covariates (not shown). a paths represent the specific indirect effect of the IV on each mediator, and b paths represent the specific indirect effect of each mediator on the DV; together, each ab pair represents the specific indirect effect of the IV on the DV through the mediator. The total indirect effect (∑ab) is the sum of all ab pairs. *Adapted from Preacher and Hayes [35]

Multiple mediation analysis was also conducted to determine if the association between having a TV in the bedroom and % BF was mediated by TV viewing time, diet patterns, MVPA, and/or sleep duration (Fig. 1). Mediation models evaluate the effect of a mediator (M) on the association between the independent variable (X) and dependent variable (Y). By convention, *a* represents the association between X and M, and *b* represents the association between M and Y while partitioning out the effect of X. The multiple mediation model described by Preacher and Hayes [35] allows one to determine the total effect of X on Y (*c*), the direct effect of X on Y (*c’*) (excluding the effects of mediators), the total indirect effect (*∑ab*) of X on Y through all of the mediators, and the effects of each mediator independent of one another and covariates (specific indirect effect, (*ab*) [35]. We used bootstrapping to assess both the total and specific indirect effects using 5000 bootstrapped samples [35]. Age, country, ethnicity, total household annual income and highest level of parental education were included as covariates in the model based on the known effect of these variables with the independent, dependent, and some mediating variables from the literature, as well as the plausibility of a potential confounding effect. The mediation effects observed were moderated by sex and country, thus the results for mediation are presented separately for boys and girls of each country. Path *a* and path *b* were considered statistically significant if p < 0.05, and path *ab* was considered statistically significant if the 95 % bias-corrected and accelerated (BCA) CI did not include zero [36].

## Results

Data were available for 1 201 children from the Canadian (n = 567) and American (n = 634) sites of ISCOLE. Canadian boys and girls with a TV in their bedroom did not differ in age or ethnicity, but were more likely to live in households with a lower annual income and lower level of parental education (Table 1). Furthermore, Canadian boys and girls with a TV in their bedroom had a higher % BF, watched more TV/day, were less likely to meet sedentary behaviour guidelines, and had unhealthier diets (Table 2).

**Table 1:** Demographic information for Canadian children by sex and presence of a TV in the bedroom

	TV in the bedroom					
	Boys			Girls		
	Yes	No	p-value^a^	Yes	No	p-value^a^
**Age (mean, SD)**	10.2 (0.4)	10.0 (0.4)	0.06	10.1 (0.37)	10.0 (0.38)	0.30
**Ethnicity [n (%), by column]**						
White/Caucasian	30 (76.9)	130 (66.3)	0.11	28 (57.1)	185 (67.0)	0.59
African American	1 (2.6)	3 (1.5)		2 (4.1)	9 (3.3)	
Asian	0	25 (12.8)		5 (10.2)	27 (9.8)	
First Nations	0	1 (0.5)		0	1 (0.4)	
East Indian	0	1 (0.5)		0	4 (1.4)	
Pacific Islander	0	0		0	0	
Don’t know	0	0		0	1 (0.4)	
Other	8 (20.5)	36 (18.4)		14 (28.6)	49 (17.8)	
**Total household annual income [n (%), by column]**						
Lowest income level	15 (40.5)	23 (12.0)	**<0.0001**	19 (38.8)	48 (18.0)	**0.003**
2^nd^ income level	10 (27.0)	50 (26.0)		16 (32.7)	76 (28.5)	
3^rd^ income level	7 (18.9)	33 (17.2)		4 (8.2)	35 (13.1)	
Highest income level	5 (13.5)	86 (44.8)		10 (20.4)	108 (40.4)	
**Highest level of parental education [n (%), by column]**						
Less than high school	0	0	**<0.0001**	1 (2.0)	1 (0.4)	**<0.0001**
Some high school	3 (7.7)	3 (1.5)		2 (4.0)	1 (0.4)	
High school diploma/GED	11 (28.0)	6 (3.1)		8 (16.0)	15 (5.4)	
Diploma or 1–3 years of college	19 (48.7)	27 (13.9)		20 (40.0)	49 (17.8)	
Bachelor's degree	4 (10.3)	73 (37.4)		10 (20.0)	85 (30.8)	
Graduate (Master's or PhD)/Professional degree	2 (5.1)	86 (44.1)		9 (18.0)	125 (45.3)	

^a^Student’s *t*-test for continuous data; Chi-square or Fisher’s exact test for categorical responses

*N = 567*

**Table 2:** Comparison of percent body fat and lifestyle behaviours between those with and without a bedroom TV among Canadian boys and girls

	Boys			Girls		
	TV in the bedroom	No TV in the bedroom	p-value^a^	TV in the bedroom	No TV in the bedroom	p-value^a^
	(n = 39)	(n = 200)		(n = 50)	(n = 278)	
**Percent body fat (mean, SD)**	21.8 (9.7)	18.1 (6.5)	**0.04**	24.9 (7.3)	21.3 (7.2)	**0.002**
**TV viewing time [hours/day category; median (IQR)]** ^**b**^	3.7 (2.4)	2.9 (1.7)	**<0.001**	4.0 (2.1)	2.7 (1.9)	**<0.0001**
≤2 h of screen time/day (n, %)	2 (5.1)	45 (22.5)	**0.01**	5 (10.0)	88 (31.7)	**0.002**
>2 h of screen time/day (n, %)	37 (94.9)	155 (77.5)		45 (90.0)	190 (68.4)	
**Healthy diet score (mean, SD)**	−0.38 (1.0)	0.04 (0.99)	**0.02**	−0.28 (0.96)	0.08 (1.0)	**0.02**
**Unhealthy diet score (mean, SD)**	0.76 (1.6)	0.06 (1.1)	**0.01**	0.18 (0.92)	−0.18 (0.76)	**0.002**
**Minutes of MVPA per day (mean, SD)**	54.1 (17.1)	59.5 (18.3)	0.11	56.2 (16.6)	59.2 (20.7)	0.36
**Sleep duration (min/night; mean, SD)**	528.4 (47.0)	541.8 (50.8)	0.15	554.9 (49.0)	547.4 (51.5)	0.36

^a^Student’s *t*-test for continuous data; Chi-square or Fisher’s exact test for categorical responses

^b^TV viewing time categories: 1 = 0 h, 2 = <1 h, 3 = 1 h, 4 = 2 h, 5 = 3 h, 6 = 4 h, and 7 = 5 or more hours of TV per day

MVPA, moderate-to-vigorous physical activity; TV, television; SD, standard deviation; IQR, inter-quartile range

*N = 567*

American boys and girls with a TV in their bedroom did not differ in age, but were more likely to live in households with a lower annual income and lower level of parental education (Table 3). A higher proportion of those children with a TV in their bedroom were African American, while a higher proportion of those children without a TV in their bedroom were Caucasian. Both American boys and girls with a TV in their bedroom watched more TV/day. Boys with a TV in their bedroom also had a higher % BF and a more unhealthy diet, while girls with a TV in their bedroom were less likely to meet sedentary behaviour guidelines and had less MVPA (Table 4).

**Table 3:** Demographic information for American children by sex and presence of a TV in the bedroom

	TV in the bedroom					
	Boys			Girls		
	Yes	No	p-value^a^	Yes	No	p-value^a^
**Age (mean, SD)**	9.6 (0.68)	9.6 (0.58)	0.89	9.5 (0.6)	9.4 (0.5)	0.10
**Ethnicity [n (%), by column]**						
White/Caucasian	65 (31.7)	45 (70.3)	**<0.0001**	80 (30.7)	65 (72.2)	**<0.0001**
African American	129 (62.9)	10 (15.5)		164 (62.8)	17 (18.9)	
Asian	1 (0.5)	6 (9.4)		9 (3.5)	6 (6.7)	
First Nations	0	0		0	0	
East Indian	0	0		0	0	
Pacific Islander	1 (0.5)	0		0	0	
Don’t know	0	0		1 (0.4)	0	
Other	9 (4.4)	3 (4.7)		7 (2.7)	2 (2.2)	
**Total household annual income [n (%), by column]**						
Lowest income level	48 (23.4)	5 (7.9)	**<0.0001**	63 (24.2)	7 (7.9)	**<0.0001**
2^nd^ income level	77 (37.6)	7 (11.1)		91 (35.3)	15 (16.9)	
3^rd^ income level	47 (22.9)	25 (39.7)		69 (26.7)	26 (29.2)	
Highest income level	33 (16.1)	26 (41.3)		35 (13.6)	41 (46.1)	
**Highest level of parental education [n (%), by column]**						
Less than high school	5 (2.4)	1 (1.6)	**<0.0001**	3 (1.1)	1 (1.1)	**<0.0001**
Some high school	17 (8.2)	1 (1.6)		23 (8.7)	3 (3.3)	
High school diploma/GED	61 (29.5)	4 (6.3)		82 (30.9)	6 (6.7)	
Diploma or 1–3 years of college	50 (24.2)	7 (10.9)		54 (20.4)	13 (14.4)	
Bachelor's degree	43 (20.8)	20 (31.3)		54 (20.4)	24 (26.7)	
Graduate (Master's or PhD)/Professional degree	31 (15.0)	31 (48.4)		49 (18.5)	43 (47.8)	

^a^Student’s *t*-test for continuous data; Chi-square or Fisher’s exact test for categorical responses

*N = 634*

**Table 4:** Comparison of percent body fat and lifestyle behaviours between those with and without a bedroom TV among American boys and girls

	Boys			Girls		
	TV in the bedroom	No TV in the bedroom	p-value^a^	TV in the bedroom	No TV in the bedroom	p-value^a^
	(n = 210)	(n = 64)		(n = 268)	(n = 92)	
**Percent body fat (mean, SD)**	21.4 (8.8)	18.9 (6.2)	**0.01**	25.1 (8.0)	24.1 (7.6)	0.30
**TV viewing time [hours/day category; median (IQR)]** ^**b**^	3.9 (3.0)	3.0 (1.7)	**<0.0001**	4.0 (2.9)	2.9 (1.5)	**<0.0001**
≤2 h of screen time/day (n, %)	21 (10.0)	10 (15.6)	0.26	48 (17.9)	28 (30.4)	**0.01**
>2 h of screen time/day (n, %)	189 (90.0)	54 (84.4)		220 (82.1)	64 (69.6)	
**Healthy diet score (mean, SD)**	−0.01 (1.72)	−0.18 (1.44)	0.48	−0.25 (2.3)	0.30 (2.4)	0.06
**Unhealthy diet score (mean, SD)**	0.14 (1.50)	−0.67 (1.91)	**0.003**	0.15 (1.5)	−0.37 (1.3)	0.05
**Minutes of MVPA per day (mean, SD)**	58.3 (20.9)	57.8 (17.4)	0.88	42.8 (15.5)	47.3 (15.6)	**0.02**
**Sleep duration (min/night; mean, SD)**	530.5 (52.3)	525.2 (41.3)	0.52	535.6 (59.9)	537.6 (53.1)	0.79

^a^Student’s *t*-test for continuous data; Chi-square or Fisher’s exact test for categorical responses

^b^TV viewing time categories: 1 = 0 h, 2 = <1 h, 3 = 1 h, 4 = 2 h, 5 = 3 h, 6 = 4 h, and 7 = 5 or more hours of TV per day

MVPA, moderate-to-vigorous physical activity; TV, television; SD, standard deviation; IQR, inter-quartile range

*N = 634*

In Canadian girls, only TV viewing time was a significant mediator of the association between having a TV in the bedroom and adiposity (Table 5). That is, the presence of a TV in the bedroom was associated with more TV viewing, which was in turn associated with higher adiposity, independent of diet patterns, MVPA and sleep duration as well as covariates. None of the lifestyle behaviours mediated the association between having a TV in the bedroom and adiposity in American girls or American and Canadian boys (data not shown).

**Table 5:** Multiple mediation analysis of the relationship between bedroom TVs and percent body fat in Canadian girls

Mediators	Path *a* estimate (SE)	Path *b* estimate (SE)	Specific indirect effect *ab* (SE)^a^	Path *ab* BCA 95 % CI^b^
TV viewing	0.70 (0.24)*	0.83 (0.35)*	0.57 (0.34)	**0.10, 1.52**
Healthy diet score	−0.23 (0.18)	−0.69 (0.45)	0.14 (0.16)	−0.05, 0.65
Unhealthy diet score	0.26 (0.14)	0.14 (0.59)	0.02 (0.22)	−0.33, 0.63
MVPA	−1.20 (3.6)	−0.02 (0.02)	0.04 (0.10)	−0.10, 0.38
Sleep duration	11.3 (9.0)	−0.01 (0.01)	−0.15 (0.17)	−0.65, 0.06

Total effect (*c*) [estimate (SE)]: 2.84 (1.27), p = 0.03

Direct effect (*c’*) [estimate (SE)]: 2.19 (1.27), p = 0.09

Total indirect effect (*∑ab*) [estimate (SE); 95 % BCA CI]: 0.62 (0.45); −0.14, 1.69

Adjusted R^2^ = 0.11, F = 3.03, p = 0.001

Adjusted for age, ethnicity, total annual household income and highest level of parental education

^*^ *p < 0.05*

^a^Path *ab* coefficients represent 5000 bootstrapped samples, bias-corrected and accelerated coefficients

^b^Estimates of *ab* path are considered significant if the BCA 95 % CI does not cross zero

SE, standard error; BCA 95 % CI, bias-corrected and accelerated 95 % confidence interval; MVPA, moderate-to-vigorous physical activity

*N =328*

## Discussion

In the current sample, 16 % and 15 % of Canadian boys and girls, as well as 77 % and 74 % of American boys and girls, had a TV in their bedroom, respectively. All children with a TV in their bedroom reported watching more TV. Canadian children and American boys with a TV in their bedroom had a higher % BF compared to those without a TV in their bedroom. Finally, Canadian children and American girls with a TV in their bedroom were less likely to obtain <2 h of screen time per day, as compared to those with a TV in their bedroom.

Effect sizes are notoriously difficult to estimate in multiple mediation [37]; however, considering that the mean difference in % BF between Canadian girls with and without a TV in their bedroom was 3.6 %, it follows that the specific indirect effect of TV viewing time was responsible for mediating ~3-42 % of variance in the difference between these groups. While this study provides the most comprehensive analysis of the potential lifestyle behaviour mediators of the association between bedroom TVs and % BF to date, the wide confidence intervals as compared to other related mediation analyses [7, 20] suggests that there is much that we still do not understand about the association between TV viewing time and adiposity in children. Residual confounding of factors included by other analyses, such as dieting history [20], is one possible explanation for these wide confidence intervals. Another explanation may be statistical power; while the current mediation analysis was conducted in a large sample of North American children, the sample was split by sex and country and thus is smaller than other previous mediation approaches [7, 20, 38].

The specificity of this finding among Canadian girls may be due to the larger difference in median hours of TV viewing per day between those with and without a TV in the bedroom. Despite relatively similar amounts of TV viewing, both Canadian and American boys were less likely to meet screen time guidelines than their female counterparts. Greater time spent playing video games or using a computer instead of watching TV may explain the null results observed in boys. Alternatively, this sex-specific effect may be due to an underpowered analysis in boys due to a smaller sample size, or residual confounding. This is also the first analysis of its kind to identify between-country differences. While this may reflect true between-country differences, children from the US site were more likely to live in a lower income household with parents who attained a lower level of education; these two factors are known to influence both TV ownership and the presence of a TV in children’s bedrooms. Neither sample is representative of the respective country and replication of these findings in nationally representative samples is warranted.

Descriptively, our results are consistent with the literature which suggests that children with a TV in their bedroom watch TV about 1 h/day more than those who do not have the same personal and ready access [5]. Likewise, this paper adds to the literature supporting the notion that children with a TV in their bedroom are at higher risk for obesity [8, 39] and cardiometabolic disease [6, 8]. However, our results are in direct contrast with the findings of Gilbert-Diamond et al. [9], who showed that the presence of a TV in the bedroom is associated with weight gain beyond the effects of TV viewing time. These authors relied on self-reported height and weight, which has been shown to be problematic in the assessment of adiposity [40], and did not examine other crucial behavioural factors associated with obesity, such as physical activity or sleep duration [9]. The use of objective measures of adiposity and lifestyle behaviours in the current analysis, as opposed to self-report measures used previously [7, 9], advances the state of the evidence for the potential mediating role of TV viewing time in the association between TV in the bedroom and adiposity, independent of other lifestyle behaviours, at least in Canadian girls.

The finding that healthy and unhealthy diet scores did not mediate the association between TV in the bedroom and adiposity is contrary to our hypothesis, and the literature [38]. There are likely other intermediate factors associated with TV viewing time and adiposity. For example, more TV viewing time provides children with more frequent opportunities for overconsumption during TV viewing [41], as well as increased exposure to TV advertisements, which have been shown to negatively affect diet patterns in children [42]. Likewise, the displacement of physical activity or sleep duration due to increased TV viewing is also a concern. In essence, more complex mediation analyses (i.e. moderated mediation, mediated moderation or multiple-step multiple mediation) are warranted to identify potential intermediate factors in the mediating role of TV viewing time and adiposity in children. For example, a recent study by Sijtsma et al. [15] showed that in Dutch pre-school children, television in the bedroom was linked with higher screen time, which was associated with decreased sleep duration, which was associated with higher BMI (a serial multiple mediation model).

Recently, the American Academy of Pediatrics [6] suggested that parents should remove TVs from children’s bedrooms. The current study provides support for this assertion. However, while health risk may increase with the presence of TV in a child’s bedroom, it is unclear if the opposite is true. Future intervention research should examine the effect of removing TVs from children’s bedrooms on their health risk over time. One intervention study by Haines et al. [43] designed to remove TVs from children’s bedrooms was unsuccessful in doing so, despite reducing BMI and TV viewing time and increasing sleep duration. If interventions continue to be unsuccessful in removing TVs from children’s bedrooms, an assessment of the perceived barriers to removing TVs will be required. It could be that these barriers are related to the reasons why parents put TVs in their children’s bedroom in the first place; namely, to keep children occupied, to help children sleep, or to free up other TVs around the house [44]. Future research should also consider other forms of media in the bedroom as well. For example, while the prevalence of TVs in the bedroom may decline over time, the prevalence of computers and tablets in the bedroom may increase [45]. In light of this, it has been suggested that parents limit the availability of all electronic entertainment and communication devices in the child’s bedroom [46].

There are several strengths of this study, including the large sample of North American children and the robust data quality assurance procedures [21]. This study is novel in that it is the first to comprehensively examine the association of TV in the bedroom with % BF, TV viewing time, diet patterns, MVPA, and sleep duration using multiple mediation analysis. Also unique to this analysis is the use of objectively measured MVPA, sedentary time, and sleep duration. There are also several limitations to the current analysis. First, although we used a large sample of children, the sample is not nationally representative of either country, and therefore results may not be generalizable. Second, the FFQ is limited in its ability to assess food intake as it does not account for food quantity or energy intake. Third, the FFQ and screen time questionnaire are subject to recall and social desirability biases; however, these measures maximize feasibility for studies with large sample sizes and reduce participant burden. Fourth, this study did not measure TV time in the bedroom specifically, which should be considered in designing future studies on the health effects of bedroom TVs. Fifthly, cross-sectional studies cannot provide information about causality and there is always the possibility of residual and incomplete confounding. As previously mentioned, the mediation model tested in the current study is based on existing evidence from longitudinal and experimental studies. Nevertheless, these results should be interpreted with caution until replicated using a longitudinal design. Finally, the school-based recruitment strategy was not considered in the current analysis because the majority of variance in adiposity was explained at the individual level, rather than the school level. While it is thought that the link between TV in the bedroom and adiposity is more strongly affected/mediated by home-based characteristics, future research should investigate the effect of school-based characteristics as well.

In conclusion, the association between TV in the bedroom and adiposity was mediated by TV viewing time, but not diet patterns, MVPA, or sleep duration, in Canadian girls only. Similar findings were not observed in Canadian boys or American girls or boys. While this study provides a comprehensive picture of the lifestyle behaviours associated with having a TV in the bedroom of a child, as well as potential mediating factors for the association with % BF, the effects of TVs in the bedroom on children’s overall health risk is far from clear. Following advice from the American Academy of Pediatrics, removing TVs from children’s bedroom has the potential to reduce TV viewing time and adiposity in future intervention studies, at least in Canadian girls.

## Abbreviations

TV: Television
MVPA: Moderate-to-vigorous physical activity
BMI: Body mass index
% BF: Percent body fat
ISCOLE: International study of childhood obesity, lifestyle and the environment
US: United States
GED: Graduate equivalent diploma
FFQ: Food frequency questionnaire
95 % BCA CI: 95 percent bias-corrected and accelerated confidence intervals

## References

[1] Strasburger VC, Jordan AB, Donnerstein E (2010). Health effects of media on children and adolescents. Pediatrics.

[2] Tremblay M, LeBlanc A, Kho M, Saunders T, Larouche R, Colley R (2011). Systematic review of sedentary behaviour and health indicators in school-aged children and youth. Int J Behav Nutr Phys Act.

[3] Maniccia DM, Davison KK, Marshall SJ, Manganello JA, Dennison BA (2011). A meta-analysis of interventions that target children’s screen time for reduction. Pediatrics.

[4] Wahi G, Parkin PC, Beyene J, Uleryk EM, Birken CS (2011). Effectiveness of interventions aimed at reducing screen time in children: a systematic review and meta-analysis of randomized controlled trials. Arch Pediatr Adolesc Med.

[5] Rideout V, Foehr U, Roberts D: Generation M2: Media in the Lives of 8- to 18-Year-Olds. Kaiser Family Foundation 2010.

[6] American Academy of Pediatrics C on CA: Children, Adolescents, and the Media. Pediatrics 2013:peds.2013–2656.

[7] Cameron AJ, van Stralen MM, Brug J, Salmon J, Bere E, Chinapaw MJM (2013). Television in the bedroom and increased body weight: potential explanations for their relationship among European schoolchildren. Pediatr Obes.

[8] Staiano AE, Harrington DM, Broyles ST, Gupta AK, Katzmarzyk PT (2013). Television, adiposity, and cardiometabolic risk in children and adolescents. Am J Prev Med.

[9] Gilbert-Diamond D, Li Z, Adachi-Mejia AM, McClure AC, Sargent JD (2014). Association of a television in the bedroom with increased adiposity gain in a nationally representative sample of children and adolescents. JAMA Pediatr..

[10] Delmas C, Platat C, Schweitzer B, Wagner A, Oujaa M, Simon C (2007). Association between television in bedroom and adiposity throughout adolescence. Obesity (Silver Spring).

[11] Demissie Z, Lowry R, Eaton DK, Park S, Kann L (2013). Electronic media and beverage intake among United States high school students–2010. J Nutr Educ Behav.

[12] O’Connor TM, Chen T-A, Baranowski J, Thompson D, Baranowski T (2013). Physical activity and screen-media-related parenting practices have different associations with children’s objectively measured physical activity. Child Obes.

[13] Cain N, Gradisar M (2010). Electronic media use and sleep in school-aged children and adolescents: a review. Sleep Med.

[14] Nuutinen T, Ray C, Roos E (2013). Do computer use, TV viewing, and the presence of the media in the bedroom predict school-aged children’s sleep habits in a longitudinal study?. BMC Public Health.

[15] Sijtsma A, Koller M, Sauer PJJ, Corpeleijn E. Television, sleep, outdoor play and BMI in young children: the GECKO Drenthe cohort. Eur J Pediatr. 2014:1–9. 10.1007/s00431-014-2443-y 25367053

[16] De Jong E, Visscher TLS, HiraSing RA, Heymans MW, Seidell JC, Renders CM (2013). Association between TV viewing, computer use and overweight, determinants and competing activities of screen time in 4- to 13-year-old children. Int J Obes (Lond).

[17] Lumeng JC, Rahnama S, Appugliese D, Kaciroti N, Bradley RH (2006). Television exposure and overweight risk in preschoolers. Arch Pediatr Adolesc Med.

[18] Epstein LH, Roemmich JN, Robinson JL, Paluch RA, Winiewicz DD, Fuerch JH (2008). A randomized trial of the effects of reducing television viewing and computer use on body mass index in young children. Arch Pediatr Adolesc Med.

[19] Robinson TN (1999). Reducing children’s television viewing to prevent obesity: a randomized controlled trial. JAMA.

[20] Carson V, Janssen I (2012). The mediating effects of dietary habits on the relationship between television viewing and body mass index among youth. Pediatric Obesity.

[21] Katzmarzyk PT, Barreira T, Broyles ST, Champagne C, Chaput J, Fogelholm M (2013). The International Study of Childhood Obesity, Lifestyle and the Environment (ISCOLE): design and methods. BMC Public Health.

[22] Barreira TV, Staiano AE, Katzmarzyk PT (2013). Validity assessment of a portable bioimpedance scale to estimate body fat percentage in white and African-American children and adolescents. Pediatr Obes.

[23] Centers for Disease Control and Prevention - YRBSS - Youth Risk Behavior Surveillance System - Adolescent and School Health. [http://www.cdc.gov/HealthyYouth/yrbs/].

[24] Schmitz KH, Harnack L, Fulton JE, Jacobs DR, Gao S, Lytle LA (2004). Reliability and validity of a brief questionnaire to assess television viewing and computer use by middle school children. J Sch Health.

[25] Lubans DR, Hesketh K, Cliff DP, Barnett LM, Salmon J, Dollman J (2011). A systematic review of the validity and reliability of sedentary behaviour measures used with children and adolescents. Obes Rev.

[26] Herman KM, Paradis G, Mathieu M-E, O’Loughlin J, Tremblay A, Lambert M. Association Between Accelerometer-Measured Physical Activity Intensities and Sedentary Time in 8–10 Year Old Children. Pediatr Exerc Sci. 2013, epub. 10.1123/pes.2012-0128 24018974

[27] Tremblay M, Leblanc A, Janssen I, Kho M, Hicks A, Murumets K (2011). Canadian sedentary behaviour guidelines for children and youth. Appl Physiol Nutr Metab.

[28] Currie C, Nic Gabhainn S, Godeau E, Roberts C, Smith R, Currie D (2008). Inequalities in Children’s Health: HBSC International Report from the 2005/2006 Survey.

[29] Vereecken CA, Maes L (2003). A Belgian study on the reliability and relative validity of the health behaviour in school-aged children food-frequency questionnaire. Public Health Nutr.

[30] Evenson KR, Catellier DJ, Gill K, Ondrak KS, McMurray RG (2008). Calibration of two objective measures of physical activity for children. J Sports Sci.

[31] Colley R, Gorber S, Tremblay M (2010). Quality control and data reduction procedures for accelerometry-derived measures of physical activity. Health Rep.

[32] Troiano RP, Berrigan D, Dodd KW, Mâsse LC, Tilert T, McDowell M (2008). Physical activity in the United States measured by accelerometer. Med Sci Sports Exerc.

[33] Tudor-Locke C, Barreira TV, Schuna JM, Mire EF, Katzmarzyk PT (2014). Fully automated waist-worn accelerometer algorithm for detecting children’s sleep-period time separate from 24-h physical activity or sedentary behaviors. Appl Physiol Nutr Metab.

[34] Barreira TV, Schuna JM, Mire E, Katzmarzyk PT, Chaput J, Leduc G (2015). Identifying children’s nocturnal sleep using 24-hour waist accelerometry. Med Sci Sports Exerc.

[35] Preacher KJ, Hayes AF (2008). Asymptotic and resampling strategies for assessing and comparing indirect effects in multiple mediator models. Behav Res Methods.

[36] Preacher KJ, Hayes AF (2004). SPSS and SAS procedures for estimating indirect effects in simple mediation models. Behav Res Methods Instrum Comput.

[37] Hayes AF (2009). Beyond Baron and Kenny: statistical mediation analysis in the new millennium. Commun Monogr.

[38] Fuller-Tyszkiewicz M, Skouteris H, Hardy LL, Halse C (2012). The associations between TV viewing, food intake, and BMI. A prospective analysis of data from the Longitudinal Study of Australian Children. Appetite.

[39] Adachi-Mejia AM, Longacre MR, Gibson JJ, Beach ML, Titus-Ernstoff LT, Dalton MA (2007). Children with a TV in their bedroom at higher risk for being overweight. Int J Obes (Lond).

[40] Shields M, Gorber S, Janssen I, Tremblay M (2011). Obesity estimates for children based on parent-reported versus direct measures. Health Rep.

[41] Chaput, Klingenberg L, Astrup A, Sjödin AM (2011). Modern sedentary activities promote overconsumption of food in our current obesogenic environment. Obes Rev.

[42] Hastings G, Stead M, McDermott L, Forsyth A, MacKintosh A, Rayner M, et al. Review of Research on the Effects of Food Promotion to Children. Final Report to the UK Food Standards Agency. University of Strathclyde; 2003.

[43] Haines J, McDonald J, O’Brien A (2013). Healthy habits, happy homes: Randomized trial to improve household routines for obesity prevention among preschool-aged children. JAMA Pediatr.

[44] Taveras EM, Hohman KH, Price S, Gortmaker SL, Sonneville K (2009). Televisions in the Bedrooms of Racial/Ethnic Minority Children: How Did They Get There and How Do We Get Them Out?. CLIN PEDIATR.

[45] Atkin AJ, Corder K (2013). Sluijs EMF van: Bedroom media, sedentary time and screen-time in children: a longitudinal analysis. Int J Behav Nutr Phys Act.

[46] Chahal H, Fung C, Kuhle S, Veugelers PJ (2013). Availability and night-time use of electronic entertainment and communication devices are associated with short sleep duration and obesity among Canadian children. Pediatr Obes.

